# Clinical characteristics of 13 cases of Coronavirus infection complicated with severe central nervous system lesions in Shanxi children’s hospital

**DOI:** 10.1186/s12890-023-02830-9

**Published:** 2024-01-04

**Authors:** Chao Du, Chaohai Wang, Fang Zhang, Xue Li

**Affiliations:** 1 Department of Pediatric Intensive Care Medicine, Shanxi’s Children Hospital, No.65, Jinxi Street, Jinyuan District, Taiyuan City, North China’s Shanxi Province 030000 China; 2 Shanxi Bethune Hospital, Shanxi Academy of Medical Sciences, Tongji Shanxi Hospital, Third Hospital of Shanxi Medical University, Taiyuan, 030032 China

**Keywords:** Novel coronavirus Infection, Omicron mutant strain, Children, Central Nervous System Disease

## Abstract

**Background:**

The new coronavirus Omicron variant strain spread rapidly worldwide and is currently the primary mutant strain prevalent in the world.

**Objective:**

To explore the clinical features of severe central nervous system lesions in children infected with novel coronavirus Omicron mutant strain, so as to provide a reference for clinical diagnosis and treatment.

**Materials and methods:**

The clinical data of 13 children diagnosed with novel coronavirus Omicron variant strain complicated with severe central nervous system infection from December 13, 2022, to January 31, 2023, in the Children’s Intensive Care Medicine Department of Shanxi Children’s Hospital were retrospectively analyzed.

**Results:**

Among the 13 children, there were 9 males (69%) and 4 females (31%); the ages ranged from 1-year-old 16 days to 13 years old, with a median age of 9 years old, and most of them were school-age children (84.6%). The 13 children were usually healthy, but this time they were all positive for the new coronavirus nucleic acid test. The 13 children had obvious signs of the abnormal nervous system when they were admitted to the hospital, among which 12 cases (92.3%) showed convulsions, 11 children had obvious disturbance of consciousness (84.6%) when they were admitted to the hospital, and 5 children had circulatory disorders (38.4%). Among the 13 children, 2 were cured (15.3%), 5 children had serious sequelae (38.4%) when they were discharged from the hospital, and 6 children died of severe illness (46.3%).

**Conclusion:**

This study illuminates the clinical characteristics of severe central nervous system complications in children with coronavirus variant infection, highlighting rapid onset, swift progression, relatively poor prognosis, and notable symptoms such as high fever, convulsions, altered consciousness, elevated interleukin-6 levels, increased cerebrospinal fluid lactate levels, and early imaging changes.

**Supplementary Information:**

The online version contains supplementary material available at 10.1186/s12890-023-02830-9.

## Background

Since the emergence of the coronavirus (COVID-19) in December 2019, the global impact of the pandemic has been profound, prompting continuous efforts to understand and address its various manifestations. The virus has exhibited an inherent capacity for evolution and mutation, resulting in the emergence of distinct variants, including Alpha, Beta, Gamma, Delta, and the most recent Omicron [1]. Notably, the Omicron (B.1.1.529) variant was first reported to the World Health Organization (WHO) by South Africa on November 24, 2021 [2], and has rapidly become the predominant mutant strain globally [3].

In the context of this evolving landscape, our study focuses on the clinical characteristics of severe central nervous system (CNS) lesions in children infected with the novel coronavirus strain [4]. As the primary variant in circulation, understanding the specific clinical features of Omicron-related CNS complications is crucial for enhancing clinical diagnosis and treatment strategies. The emergence of new variants, such as Omicron, necessitates a continuous exploration of their distinct clinical implications, contributing to the evolving body of knowledge on COVID-19 and its various presentations [5].

To augment the contextual understanding, we acknowledge the significance of exploring the long-term consequences of COVID-19-related pneumonia [6]. Additionally, insights into the radiological-pathological signatures and potential molecular pathways involved in COVID-19-related pneumomediastinum [7], contribute valuable perspectives to the broader understanding of COVID-19 complications. Furthermore, considering post-COVID-19 pulmonary complications [8], emphasizes the importance of investigating the aftermath of the infection for comprehensive patient care.

By integrating these references, we aim to provide a comprehensive background that not only addresses the novelty of our study on Omicron-related CNS lesions in children but also aligns with the broader context of COVID-19 research and its varied clinical implications.

## Objectives

This study analyzed and summarized the clinical data of children with severe central nervous system infection who were diagnosed with the new coronavirus strain in Shanxi Children’s Hospital from December 13, 2022, to January 31, 2023.

## Materials and methods

### Study design and setting

This study analyzed and summarized the clinical data of children with severe central nervous system infection who were diagnosed with the new coronavirus strain in Shanxi Children’s Hospital from December 13, 2022, to January 31, 2023.

### Participants

The study encompassed a cohort of 13 pediatric patients admitted to Shanxi Children’s Hospital, presenting with confirmed coronavirus infection and severe central nervous system (CNS) lesions. Among the participants, 9 were male, constituting 69% of the cohort, while 4 were female, accounting for 31%. The age distribution within the cohort ranged from 1 year and 16 days to 13 years, with a median age of 9 years. Notably, the majority of the participants (84.6%) belonged to the school-age demographic.

### Variables

The diagnostic criteria were referred to as “Recommendations for Early Identification, Diagnosis and Treatment of Children with Severe Coronavirus Infection” [6].

Diagnostic criteria for 2019-nCoV infection: In children with positive 2019-nCoV nucleic acid, throat swabs were used to collect pharyngeal secretions for inspection.

For clinical manifestations included:

① COVID-19-related clinical manifestations such as fever and/or respiratory symptoms; ② Significant positive signs of neurological diseases (convulsions, disturbance of consciousness, etc.); ③ In the early stage of the disease, the total number of white blood cells was normal or decreased, and the lymphocyte count was normal or decreased.

For severe and critical illness criteria included:

Diagnostic criteria for severe illness (conforming to any one of the following): (a) Hyperthermia or persistent high fever for more than 3 days; (b) Shortness of breath (< 2 months old, respiratory rate ≥ 60 times/min; 2–12 months old, respiratory rate ≥ 50 times/min; >1 ~ 5 years old, respiratory rate ≥ 40 times/min; >5 years old, respiratory rate ≥ 30 times/min), except for the influence of fever and crying; (c) At rest, when inhaling air, finger pulse oxygen saturation ≤ 0.93; (d) Nasal flaring, trident sign, wheezing; (e) Disturbance of consciousness or convulsions; (f) Refusal to eat or feeding difficulties, with signs of dehydration [6].

Diagnostic criteria for critical illness (conforming to any one of the following): (a) Respiratory failure occurs, and mechanical ventilation is required; (b) Shock occurs; (c) Combining with other organ failure requires intensive care unit (ICU) monitoring and treatment [6].

### Data sources and collection

The data collected through the electronic case of our inpatient system mainly include demographic data (gender, age, etc.), epidemiological history, basic disease history, clinical manifestations, laboratory examination data (blood routine, blood biochemical examination, cerebrospinal fluid-related tests, C-reactive protein, serum amyloid A, interleukin-6, procalcitonin, nasopharyngeal swab coronavirus nucleic acid, lymphocyte subsets, B-type natriuretic peptide, high-sensitivity troponin, blood lactic acid, blood sugar, etc.), imaging results, treatment, and outcome (Refer Supplementary Fig. 1).

The normal values of blood routine and some blood biochemical indicators of children in different age groups refer to the health industry standard implemented on October 1, 2021, “Children’s blood cell analysis reference interval” [9] and “Children’s clinical biochemical test item reference Interval” [8].

### Statistical methods

Statistical analysis was performed using SPSS 22.0 software. The measurement data with normal distribution are represented by mean ± standard deviation (xˉ±s); the measurement data with non-normal distribution are represented by median (interquartile range) [M (P25, P75)]. The count data are expressed by the number of cases and percentage (%). Non-normally distributed measurement data between the two groups was compared via the Mann-Whitney U test, and *P* < 0.05 indicated that the difference was statistically significant.

## Results

Among the 13 children, there were 9 males (69%) and 4 females (31%); the ages ranged from 1 year old 16 days to 13 years old, with a median age of 9 years old, and most of them were school-age children (84.6%). The 13 children were usually healthy, but now they were all positive for the new coronavirus nucleic acid.

All 13 children infected with coronavirus mutant strains had significant nervous system manifestations, among which 12 cases (92.3%) showed convulsions, 11 children had obvious disturbance of consciousness (84.6%) when they were admitted to the hospital, six children had abnormal cerebrospinal fluid laboratory tests (46.1%). All 13 children had high intracranial pressure on admission, and the cerebrospinal fluid pressure measured by lumbar puncture was significantly higher than normal (100%) (Table 1).

**Table 1:** The incidence of 13 cases of severe neurological symptoms in children infected with Omicron variant strains

Clinical manifestations	Number of cases	Incidence rate (%)
Convulsion	12	92.3
Disturbance of consciousness	11	84.6
Cerebrospinal fluid abnormalities	6	46.1
Manifestations of intracranial hypertension	13	100

The clinical manifestations and corresponding data include convulsions, which were observed in 92.3% of cases (12 out of 13), disturbance of consciousness in 84.6% of cases (11 out of 13), cerebrospinal fluid abnormalities in 46.1% of cases (6 out of 13), and manifestations of intracranial hypertension, which were universally present in all 13 cases, yielding a 100% incidence rate

In addition, among the 13 children, 11 cases (84.6%) had a body temperature above 39 °C on admission, the other two cases had a body temperature between 38 and 39 °C (15.4%), and five cases (38.4%) had circulatory disorders on admission. Four cases (30.8%) had obvious liver function damage on admission, and nine cases needed mechanical ventilation to maintain life (69.2%).

Among 13 children, there were viral meningitis/viral meningoencephalitis in two cases, fulminant cerebral edema in five cases, and acute necrotizing encephalopathy in six cases after diagnosis, treatment, and examination (Table 2). Two children with viral meningitis/viral meningoencephalitis aged 1 year old 16 days and 12 years old, both showed fever on admission, with body temperature higher than 39 °C and convulsions, both in the form of grand mal seizures, lumbar puncture slightly increased intracranial pressure, cerebrospinal fluid test cells slightly increased (the former was 55 × 10^6^/L, the latter was 435 × 10^6^/L). The former showed no abnormality in head imaging examination, while the latter had abnormal head magnetic resonance imaging, which showed involvement of the frontoparietal and temporal cortex in diffusion-weighted imaging (DWI). Both survived. The former recovered and was discharged after 12 days of routine hospitalization, while the latter remained mildly impaired in lower limb movement and recovered after rehabilitation.

**Table 2:** The incidence rate of 13 cases of severe nervous system disease in children infected with Omicron variant strain

Clinical manifestations	Number of cases	Incidence rate(%)
Viral neomeningitis (encephalitis)	2	15.4
Fulminant cerebral edema	5	38.5
Acute necrotizing encephalopathy	6	46.1

The clinical manifestations and corresponding data include viral neomeningitis (encephalitis) observed in 15.4% of cases (2 out of 13), fulminant cerebral edema in 38.5% of cases (5 out of 13), and acute necrotizing encephalopathy in 46.1% of cases (6 out of 13)

The prognosis of five children with fulminant cerebral edema and six children with acute necrotizing encephalopathy was poor. Two cases died within 24 h after admission, and six cases died within one week. Two of the five children with fulminant cerebral edema died within 24 h of admission. Brain herniation occurred in one case, who died of multi-organ failure one week after admission. The remaining two cases survived after treatment but left significant sequelae. Four of the five children had convulsions when they were admitted to the hospital, which manifested as grand mal seizures and the children with brain herniation did not have convulsions. All five children except the one with cerebral hernia had a fever, and their body temperature exceeded 39 °C. All five children were in a deep coma when they were admitted to the hospital, and the Glasgow coma score was less than five. Two of the five children had circulatory disturbances, and four of them required mechanical ventilation (Table 3).

**Table 3:** Main manifestations of five cases of Omicron mutant infection complicated with fulminant cerebral edema

Clinical manifestations	Number of cases	Incidence rate(%)
Convulsion	4	80
Deep coma	5	100
High fever (Exceed 39℃)	4	80
Circulation disorder	2	40
Mechanical Ventilation	4	80

The clinical manifestations and corresponding data include convulsions, observed in 80% of cases (4 out of 5), deep coma universally present in all cases (100%), high fever exceeding 39℃ in 80% of cases (4 out of 5), circulation disorder in 40% of cases (2 out of 5), and the need for mechanical ventilation in 80% of cases (4 out of 5)

There were obvious abnormalities in the laboratory tests of the five children, mainly manifested as a decrease in the absolute value of lymphocytes, an increase in serum amyloid, an increase in interleukin 6, and an increase in the level of lactic acid in the cerebrospinal fluid. (The lumbar puncture for children with cerebral herniation is to re-examine the imaging examination five days after admission to check that the brain herniation has not been reset. After evaluating that the examination has no adverse relationship with the prognosis of the child, communicate with the family members of the child and agree to perform the examination.) (Table 4).

**Table 4:** The main laboratory indicators and prognosis of five children with Omicron variant infection complicated with fulminant cerebral edema

Parameter	Grade	Number of cases	Number of deaths	Number of deaths, mean ± SD
Absolute value of lymphocytes	Elevated or normal	1	1	0.20 ± 0.45
	Reduced	4	2	0.40 ± 0.55
Serum amyloid (SAA)	Raised	5	3	0.60 ± 0.55
	Reduced or normal	0	0	0.00 ± 0.00
C-reactive protein (CRP)	Raised	2	1	0.20 ± 0.45
	Reduced or normal	3	2	0.40 ± 0.55
Procalcitonin (PCT)	Raised	4	2	0.40 ± 0.55
	Mild elevated or normal	1	1	0.20 ± 0.45
Interleukin-6	Raised	5	3	0.60 ± 0.55
	Reduced or normal	0	0	0.00 ± 0.00
Cerebrospinal fluid cell count	Raised	2	2	0.40 ± 0.55
	Reduced or normal	3	1	0.20 ± 0.45
Cerebrospinal fluid lactic acid	Raised	5	3	0.60 ± 0.55
	Reduced or normal	0	0	0.00 ± 0.00
Cerebrospinal fluid lactate dehydrogenase	Raised	5	3	0.60 ± 0.55
	Reduced or normal	0	0	0.00 ± 0.00
D-2mer	Raised	3	3	0.60 ± 0.55
	Reduced or normal	2	0	0.00 ± 0.00

This table provides a comprehensive insight into the laboratory indicators associated with fulminant cerebral edema in children infected with the Omicron variant, along with the corresponding prognostic outcomes

**Fig. 1:**
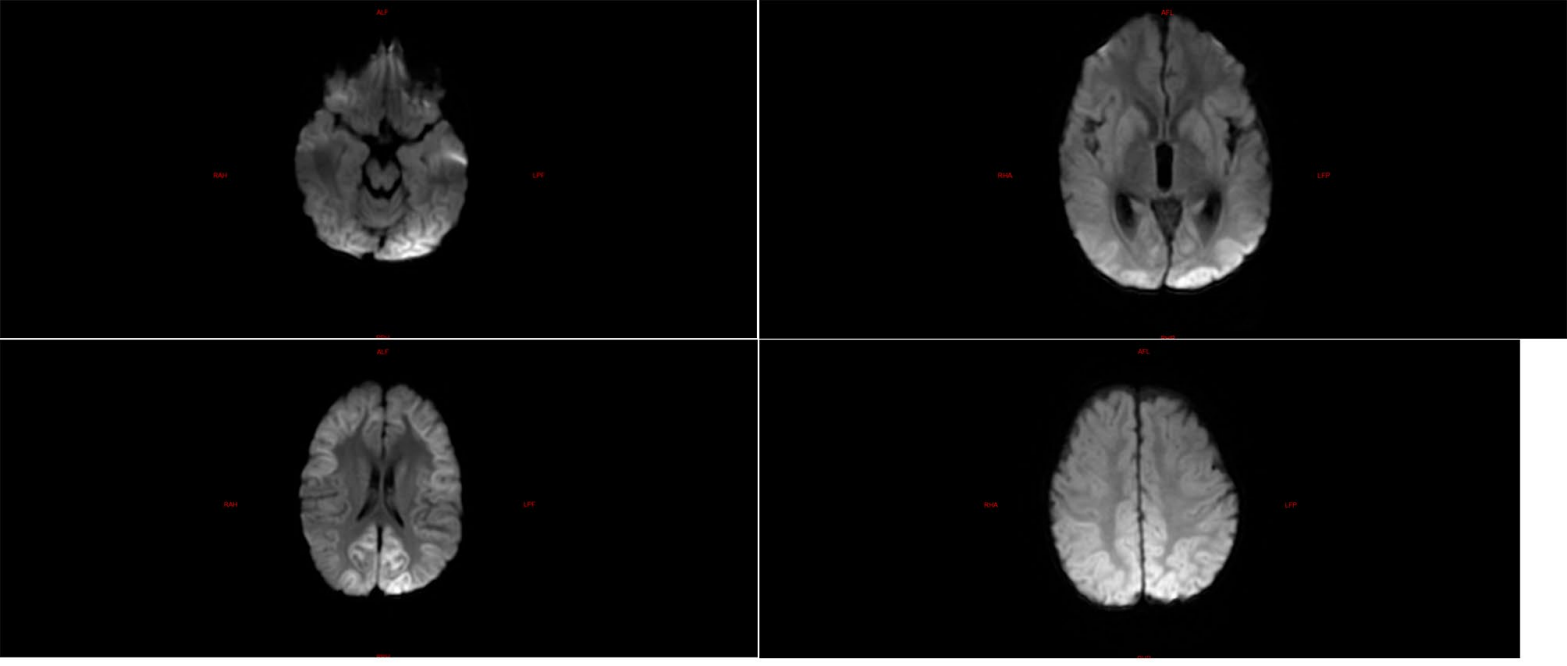
**a**, **b**, **c**, and **d** are brain MR DWI sequences of case 1, showing symmetrical diffuse hyperintensities in both supratentorial cerebral hemispheres, mainly involving the cortex and the basal ganglia. The patient was ultimately diagnosed with fulminant cerebral edema, with a rapid decline in consciousness to a GCS score of 3 within a short period. Despite stabilizing vital signs through aggressive treatment, the patient ultimately developed severe cognitive impairment and residual sequelae of limb paralysis

**Fig. 2:**
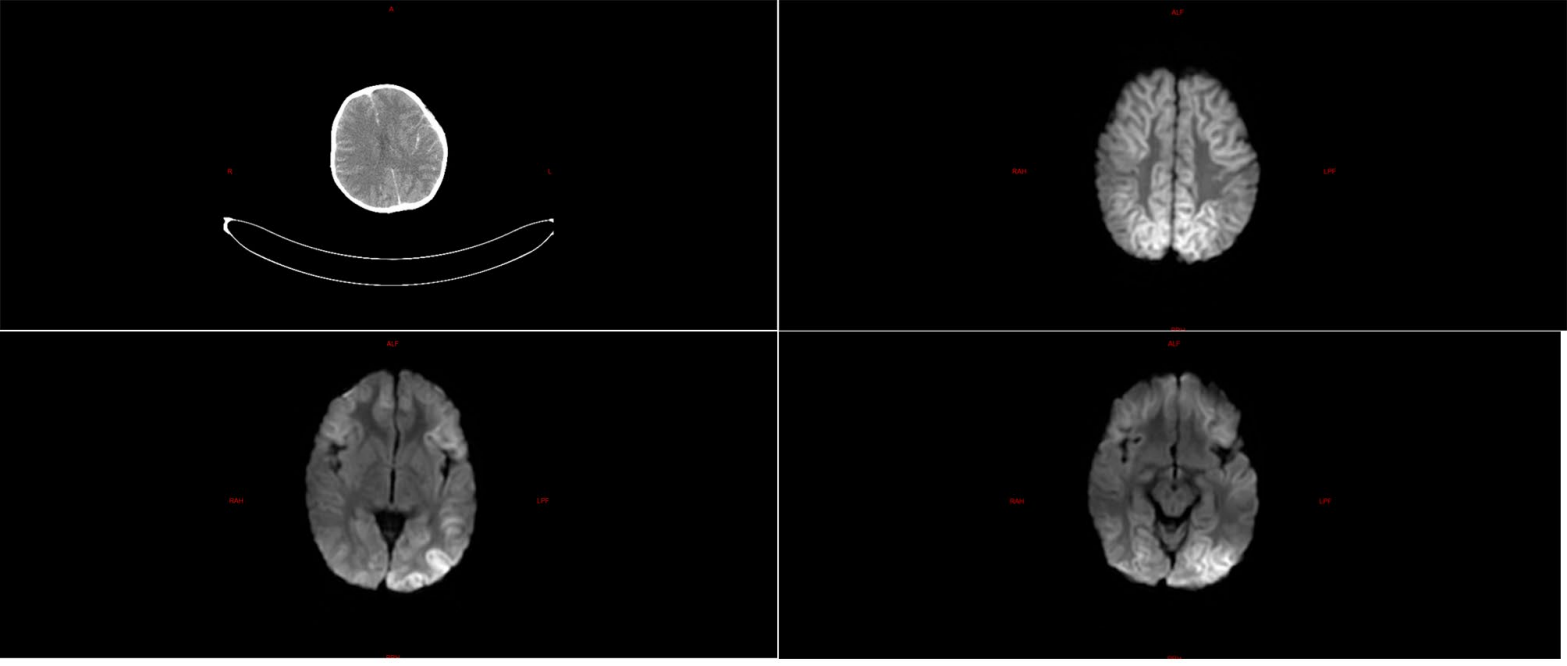
**a**: Enhanced brain CT image of case 2, showing diffuse swelling of bilateral cerebral hemispheres, shallower sulci, decreased cortical density, and linear enhancement in bilateral temporal lobe sulci; **b**, **c**, and **d** are brain MR DWI sequences of case 2, showing symmetrical diffuse hyperintensities in both cerebral hemispheres, mainly involving the cortex. The patient experienced a rapid deterioration of condition shortly after admission, manifesting cortical stiffness within a short timeframe and developing central diabetes insipidus. Unfortunately, the patient succumbed to multiple organ failure in the end

**Fig. 3:**
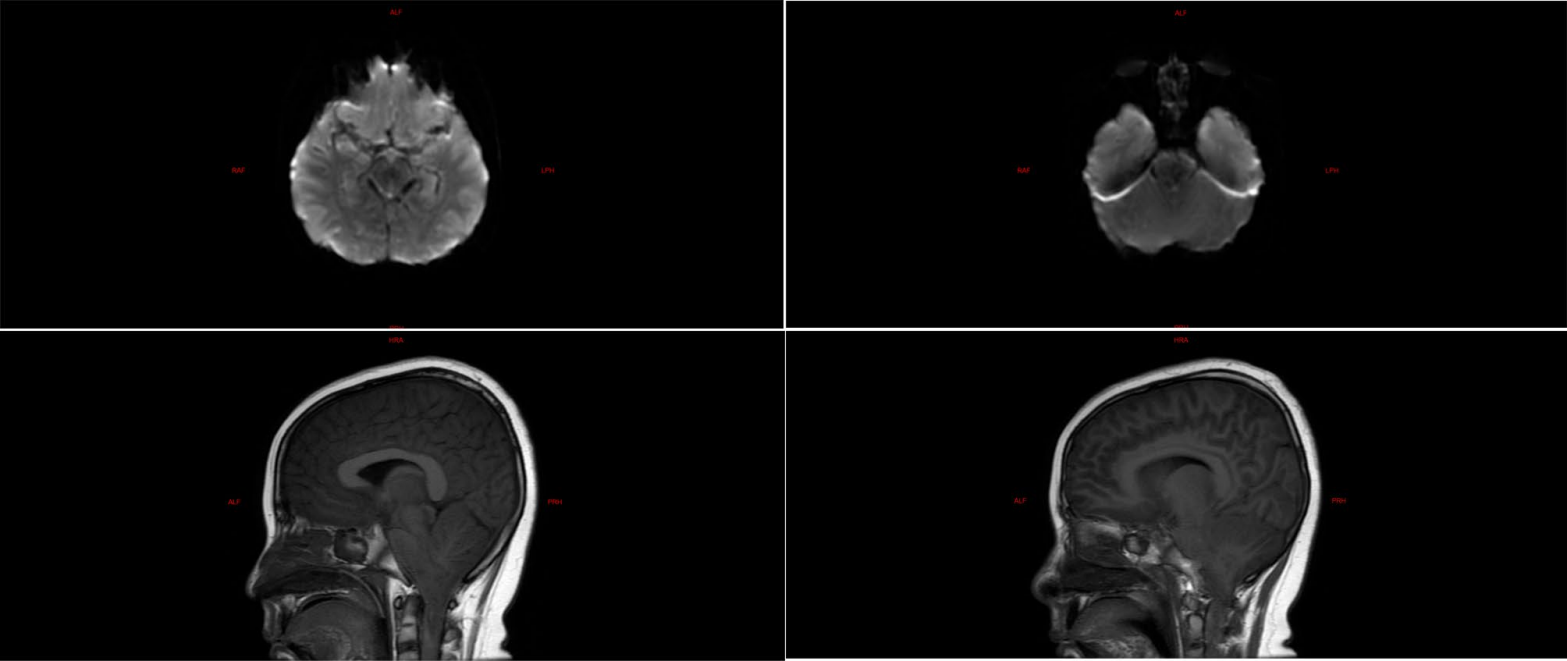
Brain MR images of case 3. **a**, **b** are DWI sequences, showing bilateral temporal and occipital lobes symmetrical diffuse high signal; **c**, **d** are T1 WI sagittal sequences, showing cerebellar sub-tonsillar herniation. The patient developed herniation of the brain induced by fulminant cerebral edema, exhibiting rapidly progressing decerebrate rigidity shortly after admission, accompanied by central diabetes insipidus. Unfortunately, the patient passed away after the decision to discontinue treatment

Among the five children with fulminant cerebral edema, three children underwent brain imaging examination after admission, and the other two died within 24 h due to critical condition after admission, without imaging examination. Among them, three children had significant abnormalities in cranial imaging examinations, one child had cerebral herniation, and the other two children had significant DWI abnormalities (Refer Figs. 1, 2 and 3).

Among the six children with acute necrotizing encephalopathy, one died of multiple organ failure, two died after their parents gave up treatment and were extubated, and the other three survived, but all left severe disabilities. Five of the six children had convulsions when they were admitted to the hospital, and the manifestation was grand mal seizures. All six children had severe disturbance of consciousness, and the Glasgow coma score was less than five. All six children had a fever, and five cases had body temperatures over 39 °C. Four of the six children required mechanical ventilation. There were circulatory disorders in two patients (Table 5).

**Table 5:** Main manifestations of six cases of Omicron mutant infection complicated with acute necrotizing encephalopathy

Clinical manifestations	Number of cases	Incidence rate(%)
Convulsion	5	83.3
Deep coma	6	100
High fever (Exceed 39℃)	5	83.3
Circulation disorder	2	33.3
Mechanical Ventilation	4	66.7

The clinical manifestations and corresponding data include convulsions, observed in 83.3% of cases (5 out of 6), deep coma universally present in all cases (100%), high fever exceeding 39℃ in 83.3% of cases (5 out of 6), circulation disorder in 33.3% of cases (2 out of 6), and the need for mechanical ventilation in 66.7% of cases (4 out of 6)

**Fig. 4:**
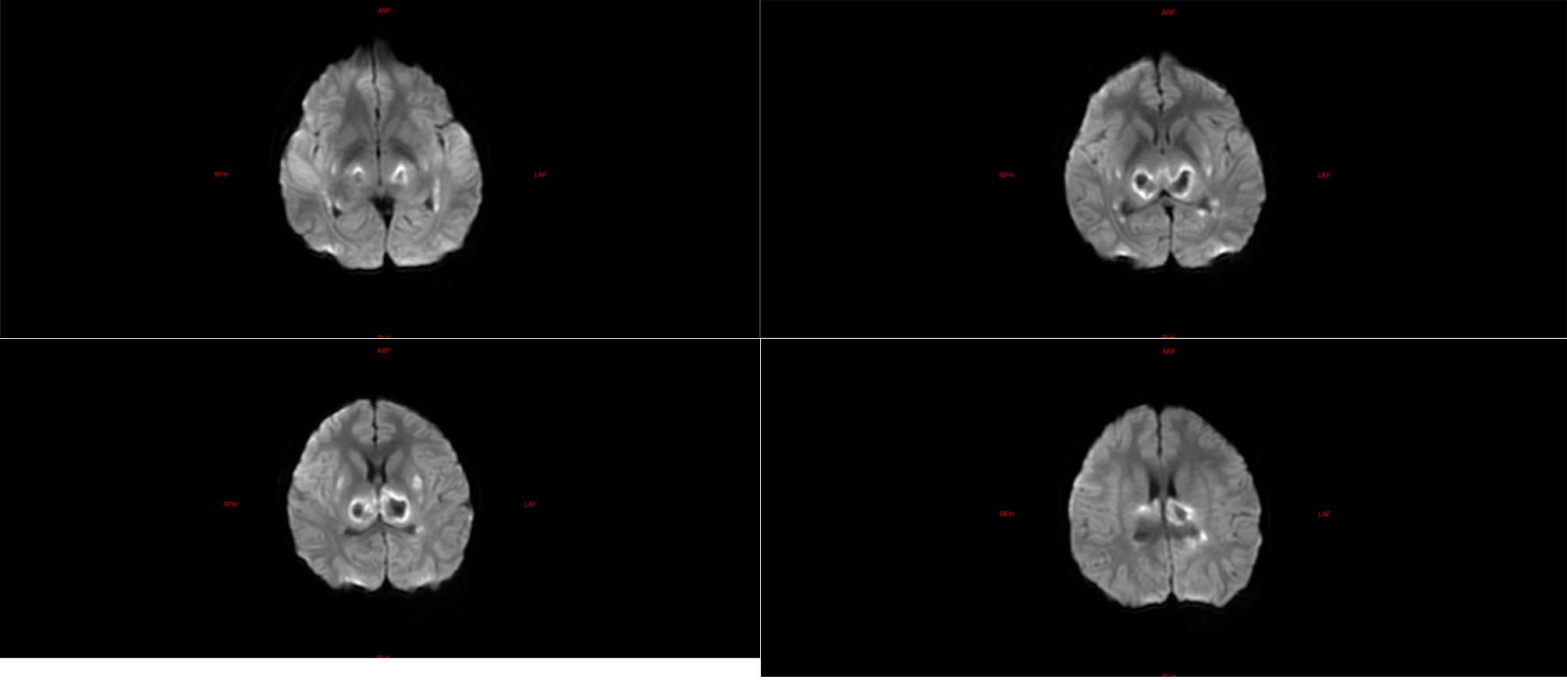
Brain MR DWI sequence of case 4, showed bilateral basal ganglia, thalamus, and white matter adjacent to the posterior horn of the lateral ventricle with symmetrical diffuse high signal, and a low signal was seen in the bilateral thalamic lesions, and hemorrhage was considered. The patient presented with typical features of necrotizing encephalopathy, exhibiting significant consciousness impairment and irregular respiratory rhythm upon admission. Following treatment, the patient was transferred from the ICU, but later experienced cognitive regression and impairment in motor function

**Fig. 5:**
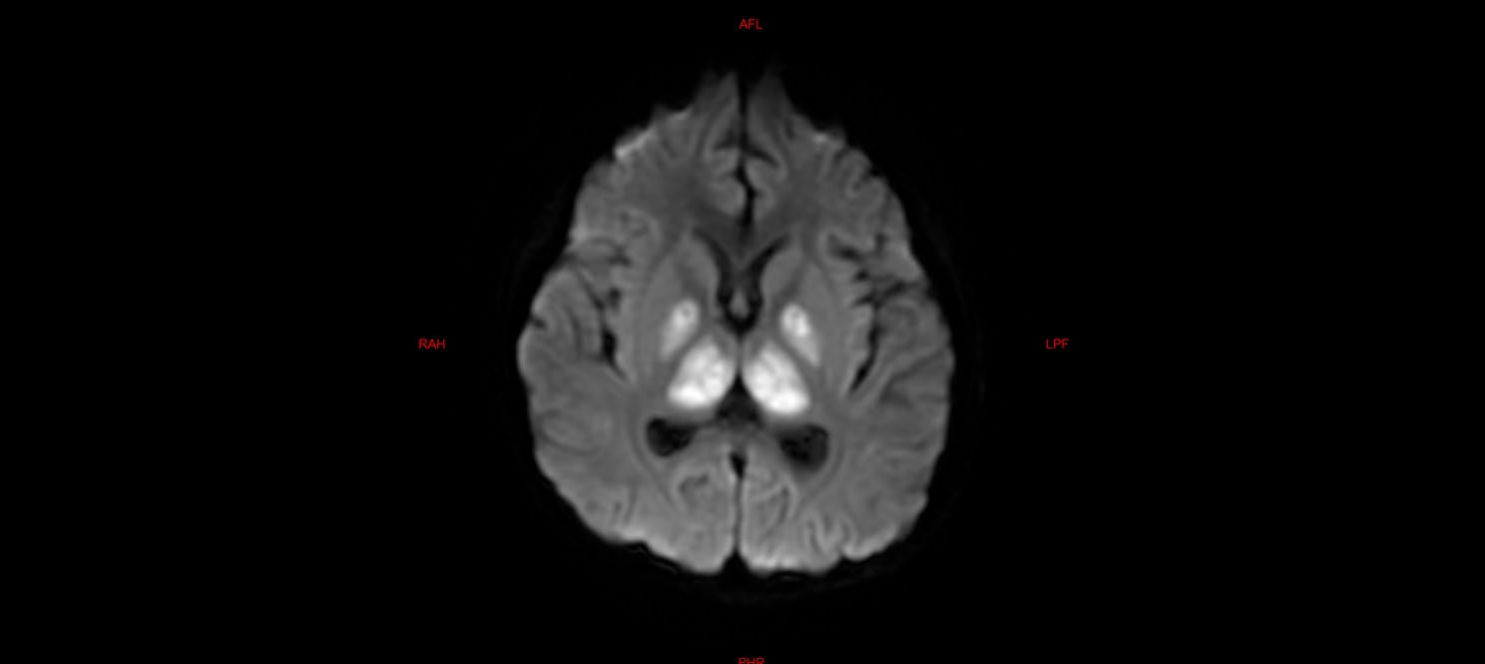
Brain MR DWI sequence of case 5, showing symmetrical diffuse high signal in bilateral basal ganglia and thalamus. The patient was diagnosed with necrotizing encephalopathy. Shortly after admission, there was a rapid deepening of consciousness impairment, leading to cerebral dysfunction, cessation of spontaneous breathing, and concomitant circulatory failure. Ultimately, the patient succumbed to multiple organ failure

**Fig. 6:**
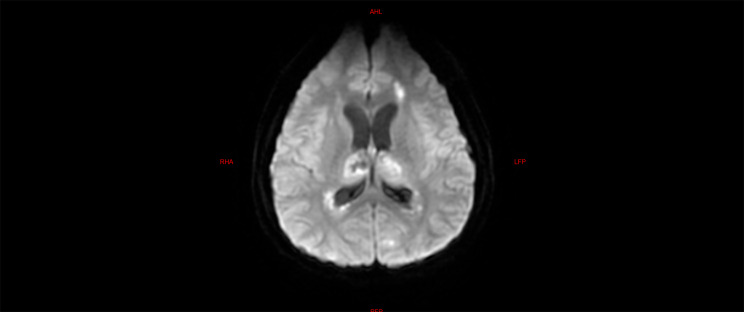
Brain MR DWI sequence of case 6, showed a patchy high signal in the bilateral thalamus and periventricular white matter, and a low signal in the right thalamic lesion, considering bleeding. The patient was diagnosed with necrotizing encephalopathy. Following admission, there were complications of central respiratory failure and circulatory failure, necessitating basic life support through mechanical ventilation and high-dose vasoactive drugs. Despite treatment, there was no improvement, and ultimately, the parents chose to discontinue treatment, leading to the patient’s demise

**Fig. 7:**
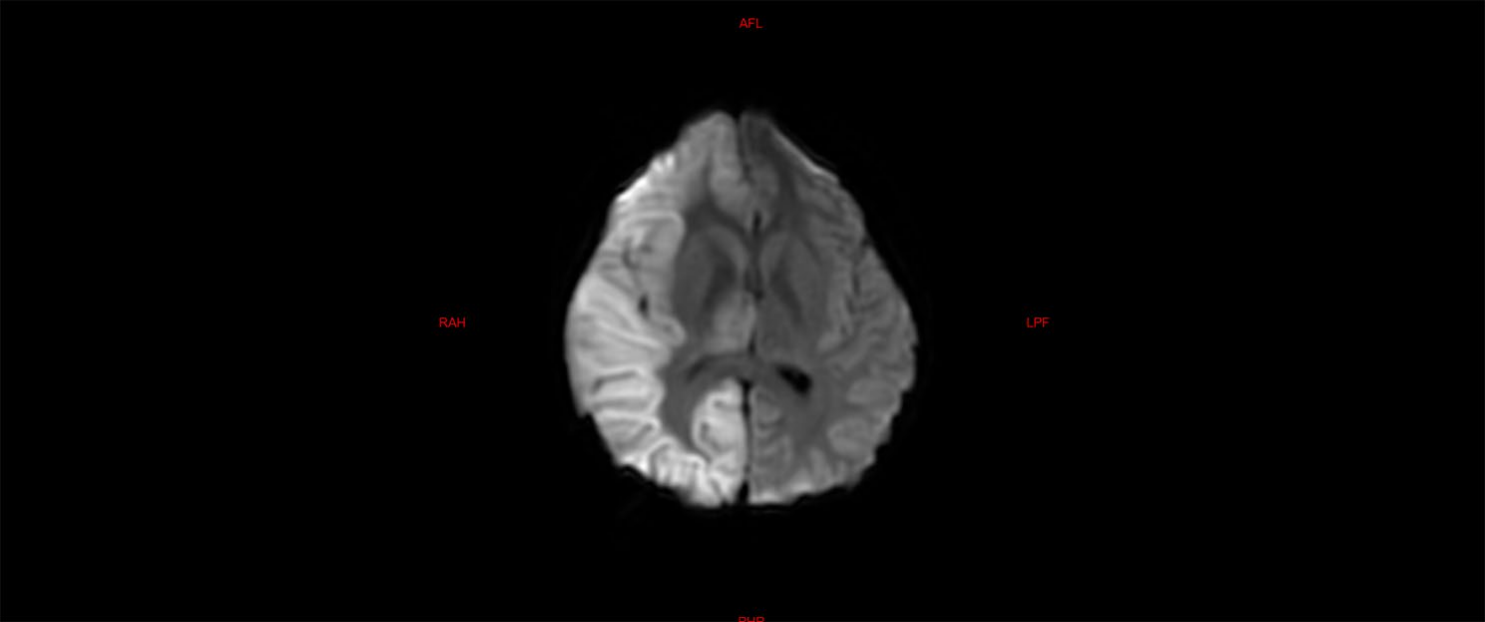
Brain MR DWI sequence of case 7, showing diffuse swelling of the right cerebral hemisphere, shallow sulcus fissure, diffuse diffuse high signal, patchy diffuse high signal in the right basal ganglia and thalamus, and pressure change of the right lateral ventricle Flattened, the midline structure is slightly shifted to the left. Upon admission, the patient developed edema in the right hemisphere of the brain, along with damage to the right basal ganglia and thalamus. Despite treatment, necrotizing encephalopathy was still considered. Although the patient was successfully weaned off advanced life support, severe motor and cognitive impairments persisted

**Fig. 8:**
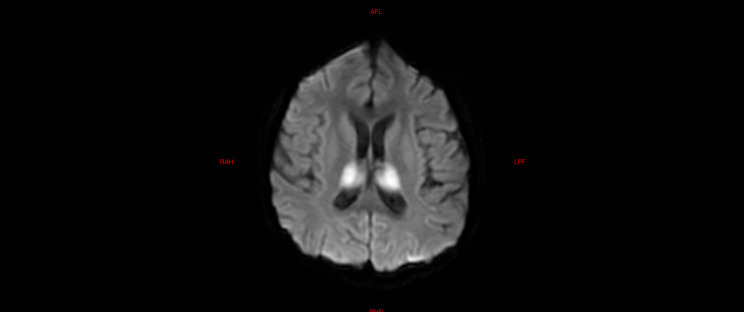
Brain MR DWI sequence of case 8, showing symmetrical diffuse high signal in bilateral thalamus. The patient, considered to have necrotizing encephalopathy, exhibited pronounced consciousness impairment upon admission, temporarily experiencing concomitant circulatory and respiratory failure. Following treatment, the patient was successfully weaned off advanced life support; however, there persisted severe cognitive regression and motor dysfunction

**Fig. 9:**
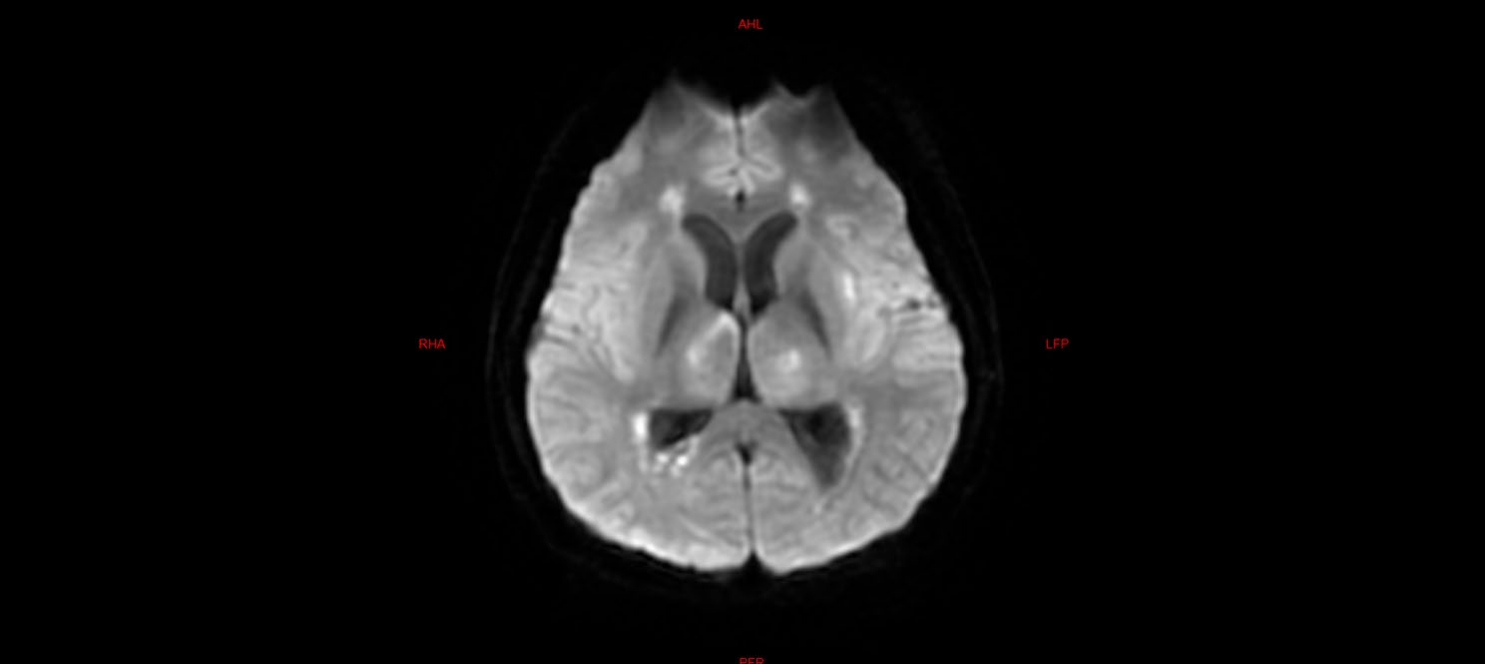
Brain MR DWI sequence of case 9, showed multiple patchy diffuse hyperintensities in the left basal ganglia, bilateral thalamus, and bilateral lateral ventricle white matter. The patient, suspected to have necrotizing encephalopathy, presented in critical condition upon admission, with concurrent multiple organ failure and central diabetes insipidus. Subsequently, due to financial constraints, the family opted to discontinue treatment, leading to the patient’s demise

The laboratory tests of the six children were similar to those of the five children with fulminant cerebral edema. The main manifestations were a decrease in the absolute value of lymphocytes, an increase in serum amyloid, an increase in interleukin-6, and an increase in lactic acid in the cerebrospinal fluid (Table 6). All six children with acute necrotizing encephalopathy had significantly abnormal cranial imaging findings (Refer Figs. 4, 5, 6, 7, 8 and 9).

**Table 6:** Main laboratory indicators and prognosis of six children with Omicron variant infection complicated with acute necrotizing encephalopathy

Parameter	Grade	Number of cases	Number of deaths	Number of deaths, mean ± SD
Absolute value of lymphocytes	Elevated or normal	0	0	0.00 ± 0.00
	Reduced	6	0	0.00 ± 0.00
Serum amyloid (SAA)	Raised	6	3	0.50 ± 0.55
	Reduced or normal	0	0	0.00 ± 0.00
C-reactive protein (CRP)	Raised	2	1	0.17 ± 0.41
	Reduced or normal	4	2	0.33 ± 0.52
Procalcitonin (PCT)	Raised significantly	3	1	0.17 ± 0.41
	Mild elevated or normal	3	2	0.33 ± 0.52
Interleukin-6	Raised	6	3	0.50 ± 0.55
	Reduced or normal	0	0	0.00 ± 0.00
Cerebrospinal fluid cell count	Raised	2	1	0.17 ± 0.41
	Reduced or normal	4	2	0.33 ± 0.52
Cerebrospinal fluid lactic acid	Raised	6	3	0.50 ± 0.55
	Reduced or normal	0	0	0.00 ± 0.00
Cerebrospinal fluid lactate dehydrogenase	Raised	3	2	0.33 ± 0.52
	Reduced or normal	3	1	0.17 ± 0.41
D-2mer	Raised	3	1	0.17 ± 0.41
	Reduced or normal	3	2	0.33 ± 0.52

This table offers a comprehensive insight into the laboratory indicators associated with acute necrotizing encephalopathy in children infected with the Omicron variant, along with the corresponding prognostic outcomes

## Discussion

The findings of this study shed light on the clinical characteristics of children with coronavirus variant infection complicated by severe central nervous system (CNS) lesions, though it is essential to acknowledge the limitations associated with the absence of controlled studies. In comparing our results with a multi-center study in South Africa [10], it is notable that COVID-19-related hospitalizations during the epidemic of the coronavirus variant were reportedly lower than in previous waves. However, a significant increase in hospitalizations among children and adolescents was observed, suggesting a potentially heightened transmissibility of the coronavirus variant in this demographic. It is crucial to interpret these observations cautiously, considering the evolving nature of the pandemic and the lack of controlled studies to establish definitive causal relationships. As life returns to normal and the number of children infected with the coronavirus mutant strain rises, it becomes imperative to address the challenges in early identification and treatment, given the increasing severity of cases, particularly those involving severe central nervous system complications induced by the variant. Early identification and treatment of these children pose significant challenges, with associated high economic costs. Based on the cases gathered in this study, these children exhibited abrupt onset, swift progression, a heightened risk of adverse prognoses, and posed challenges in terms of treatment. Thus, there is a pressing need for heightened awareness and attention to the early identification of such cases.

Despite the limited sample size of only 13 patients in this study, certain shared clinical characteristics among children with coronavirus variant infection and severe central nervous system complications can be observed. The initial presentations of severe neurological diseases following infection with the coronavirus variant strain predominantly included high fever, convulsions (the incidence of convulsions upon admission to the ICU ranges from 0 to 3 per day), and disturbances of consciousness, as noted in the majority of the 13 children under investigation. Utilizing the Glasgow Coma Scale (GCS), the patients’ neurological status is evaluated through a combination of eye (E), verbal (V), and motor (M) responses. The GCS scores range from E1TM1 to E4V3M6, providing a detailed assessment of the patients’ level of consciousness and central nervous system involvement in the context of coronavirus infection (Refer Supplementary Table 1). Therefore, post-infection vigilance should be directed towards monitoring mental states, consciousness, and temperature changes, prompting timely medical intervention when these indicators arise. Children receiving hospital treatment warrant close attention to their laboratory parameters and imaging examinations.

In this study, elevated levels of interleukin-6 were significantly noted in all cases involving fulminant cerebral edema and acute necrotizing encephalopathy. Particularly, deceased children (six dead children) exhibited substantially higher interleukin-6 levels compared to surviving counterparts. This suggests a potential association between coronavirus mutant strains and a surge in inflammatory factors, culminating in irreversible damage to organs and tissues. Speculation on the potential efficacy of early interventions such as plasma exchange or the adsorption of plasma inflammatory factors in reducing mortality and disability rates requires further confirmation through rigorous investigation. Regarding other infection indicators, the specificity of serum amyloid (SAA) in the early stages of infection was notably higher than that of C-reactive protein and procalcitonin. Attention should be directed to the early elevation of D-2mer levels, which may be indicative of a poor prognosis. Early cerebrospinal fluid examination is deemed essential, with increased lactic acid levels in the early stages suggesting compromised neuronal metabolism and signaling a poor prognosis.

Regarding treatment modalities, the early administration of tocilizumab combined with high-dose intravenous human immunoglobulin pulse therapy shows promise in achieving a high survival rate, two of the five children with fulminant cerebral edema received the tocilizumab treatment had survived. Three of the six children with necrotizing encephalopathy were treated with tocilizumab, and one of them died. All five children with fulminant cerebral edema and six children with acute necrotizing encephalopathy received intravenous injections of human immunoglobulin (2 g/Kg). However, the limited sample size warrants caution in drawing definitive conclusions. The role of glucocorticoids remains uncertain, and further investigation is essential to delineate their potential benefits and risks. Early imaging examinations within 48 h of admission facilitate the clinical identification of critical illnesses. While our study underscores the value of early imaging, acknowledging the need for larger-scale controlled studies is essential to validate these findings.

In summary, this study contributes valuable insights into the clinical characteristics of severe central nervous system diseases in children with coronavirus variant infection as characterized by rapid onset, swift progression, relatively poor prognosis, and significant early symptoms. These include high fever, convulsions, altered consciousness, elevated interleukin-6 levels, increased cerebrospinal fluid lactate levels, and early imaging changes. However, the limitations associated with the lack of controlled studies underscore the need for continued research to establish more robust conclusions regarding the management and outcomes of such cases.

### Limitations of the study

A few areas for improvement are found in this study. First, this study is a single-center retrospective case study in the Department of Pediatric Intensive Care Medicine, Shanxi Children’s Hospital. The sample size of the study is small and has limitations. In future studies, more sample size which involves participants from other hospitals is highly recommended. Second, this study only involved children subjects. Clinical manifestations of adults infected with the coronavirus variant complicated with severe central nervous system lesions are not yet determined.

## Conclusion

While our study sheds crucial light on the clinical characteristics of severe central nervous system diseases in children with coronavirus variant infection, emphasizing the rapid onset, swift progression, and notable early symptoms, it is imperative to acknowledge the inherent limitations stemming from the absence of controlled studies. The findings underscore the urgency of heightened awareness, early identification, and prompt treatment in the face of increased severity, particularly in cases involving severe central nervous system complications induced by the variant. As the pandemic evolves and the prevalence of the coronavirus mutant strain rises among children, the challenges in identifying and managing these cases become increasingly evident. In essence, our study underscores the need for ongoing research to establish more robust conclusions and effective strategies for the identification, treatment, and reduction of mortality and disability rates associated with severe central nervous system complications in children with coronavirus variant infection.

### Electronic supplementary material

Below is the link to the electronic supplementary material.

Supplementary Table 1: Demographic Information and Glasgow Coma Scale (GCS) Scores of Patients upon Admission

Supplementary Figure 1: Overview of Data sources and collection

## Data Availability

The datasets used and/or analysed during the current study available from the corresponding author on reasonable request.
