# Supplementary material for: Clinical characteristics of 13 cases of Coronavirus infection complicated with severe central nervous system lesions in Shanxi children’s hospital
Source: BMC Pulm Med. 2024 Jan 4;24:12. doi: 10.1186/s12890-023-02830-9 (PMC10768453; doi:10.1186/s12890-023-02830-9)
Supplement: Supplementary file 1 — Supplementary Table 1: Demographic Information and Glasgow Coma Scale (GCS) Scores of Patients upon Admission [file 12890_2023_2830_MOESM1_ESM.docx]

| Supplementary Table 1. Demographic Information and Glasgow Coma Scale (GCS) Scores of Patients upon Admission | | |
| --- | --- | --- |
| Gender | Age | Admission Consciousness, GCS Score |
| Male | 3 years | E1V1M5 |
| Female | 8 years | E1V1M2 |
| Male | 7 years | E1V1M2 |
| Female | 9 years | E2V1M4 |
| Female | 6 years | E1V1M1 |
| Male | 10 years | E2TM4 |
| Female | 1 year 1 month | E1TM1 |
| Female | 10 years | E1V1M1 |
| Male | 13 years | E3V2M5 |
| Male | 8 years | E1V2M2 |
| Male | 1 month 16 days | E2V2M2 |
| Male | 12 years | E4V3M6 |
| Male | 11 years | E2V1M5 |

This table provides an overview of the demographic details, including gender and age, along with the admission consciousness status assessed using the Glasgow Coma Scale (GCS) scores for each patient upon admission to the hospital.
