# Supplementary figures and images for: Clinical characteristics of 13 cases of Coronavirus infection complicated with severe central nervous system lesions in Shanxi children’s hospital
Source: BMC Pulm Med. 2024 Jan 4;24:12. doi: 10.1186/s12890-023-02830-9 (PMC10768453; doi:10.1186/s12890-023-02830-9)

Supplementary Figure 1. Overview of Data sources and collection

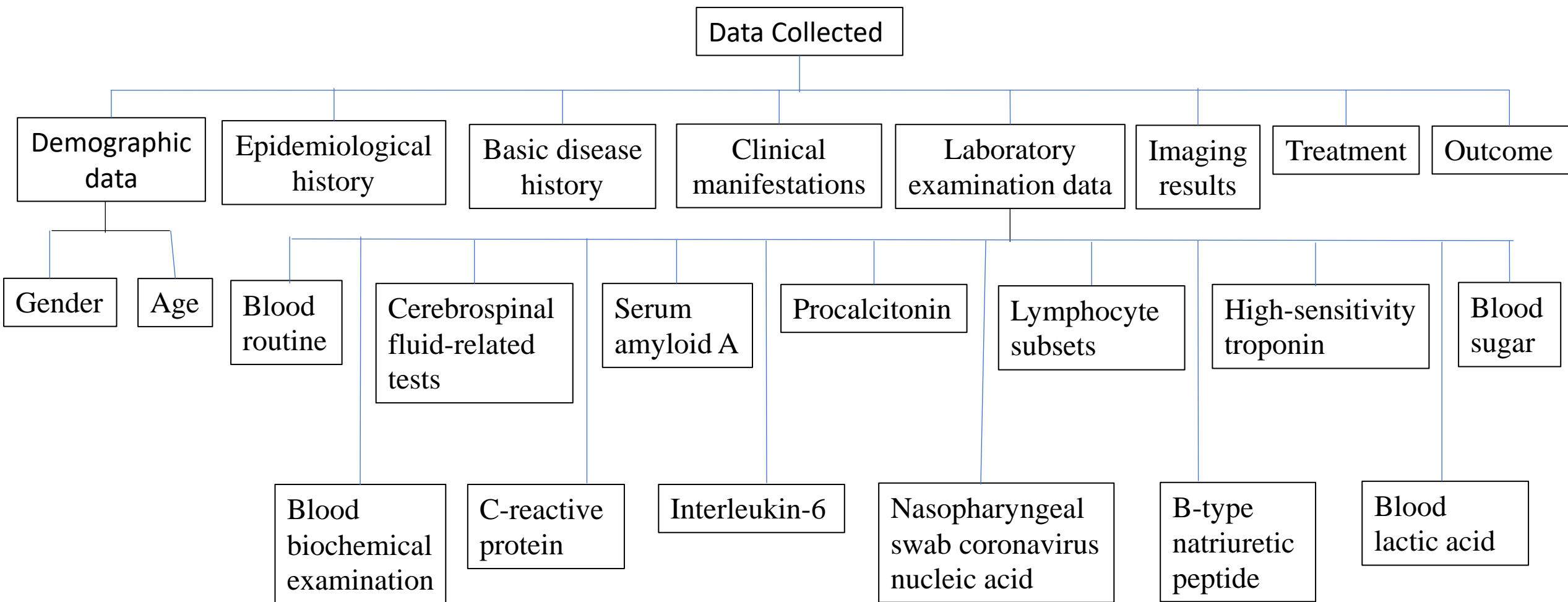

Supplement: Supplementary file 2 — Supplementary Figure 1: Overview of Data sources and collection [file 12890_2023_2830_MOESM2_ESM.pdf]
